# Is atopic sensitization associated with indicators of early vascular ageing in adolescents?

**DOI:** 10.1371/journal.pone.0220198

**Published:** 2019-08-15

**Authors:** Karsten Königstein, Denis Infanger, Randi Jacobsen Bertelsen, Ane Johannessen, Ulrike Waje-Andreassen, Arno Schmidt-Trucksäss, Cecilie Svanes, Julia Dratva

**Affiliations:** 1 Department of Sport, Exercise and Health, Division Sports and Exercise Medicine, University of Basel, Basel, Switzerland; 2 Department of Clinical Science, University of Bergen, Bergen, Norway; 3 Department of Occupational Medicine, Haukeland University Hospital, Bergen, Norway; 4 Centre for International Health, Department of Global Public Health and Primary Care, University of Bergen, Bergen, Norway; 5 Department of Neurology, Haukeland University Hospital, Bergen, Norway; 6 Medical Faculty, University of Basel, Basel, Switzerland; 7 ZHAW, School of Health Professions, Winterthur, Switzerland; Medical University Innsbruck, AUSTRIA

## Abstract

**Background:**

Chronic systemic inflammation accelerates early vascular ageing. Atopic sensitization and allergic diseases may involve increased inflammatory activity. This study aimed to assess whether atopic sensitization and allergic diseases were associated with altered vascular biomarkers in Norwegian adolescents.

**Methods:**

Distensibility coefficient of the common carotid arteries, carotid intima-media thickness and atopic sensitization (serum total and specific IgEs) were assessed in 95 Norwegian adolescents, who participated in the RHINESSA generation study. Symptoms of allergic disease were assessed by an interviewer-led questionnaire.

**Results:**

Atopic sensitization was found in 33 (34.7%) of the adolescents. Symptomatic allergic disease was found in 11 (33.3%) of those with atopic sensitization. Distensibility coefficient of the common carotid arteries appeared to be lower in participants with atopic sensitization than in those without (46.99±8.07*10^−3^/kPa versus 51.50±11.46*10^−3^/kPa; *p*>0.05), while carotid intima-media thickness did not differ between these groups (0.50±0.04mm versus 0.50±0.04mm; *p*>0.05). Crude, as well as age- and sex-adjusted multiple regression, revealed no significant association, neither of atopic sensitization nor of allergic disease, with distensibility coefficient of the common carotid arteries and carotid intima-media thickness.

**Conclusions:**

Our results do not support the assumption of an adverse impact of atopic sensitization and/or allergic disease on distensibility coefficient of the common carotid arteries and carotid intima-media thickness in Norwegian adolescents. Further research is necessary to study whether the clinical severity of allergic diseases might be more important than the status of allergic disease or atopic sensitization.

## Introduction

Evidence is growing, that inflammatory processes play an important role in atherogenesis, promoting the risk of cardiovascular diseases [1]. Possible pathophysiological links between inflammation and vascular damage were previously described [2–8]. One of the most investigated mechanisms is the oxidative modification of LDL, which leads to foam cell formation and development of lesions in the vascular wall [2, 3]. Wang et al. observed a stimulated arterial cell apoptosis and cytokine expression in humans and mice by elevated serum IgE levels [4]. This might be preceded by decreased serum-levels of low-affinity IgE receptor-positive B cells, as observed after coronary artery bypass graft surgery [5]. A possible link between chronic inflammatory activity caused by atopic sensitization and atherosclerosis might be an elevated activity of mast cells, which leads to multiple effects in the vascular wall, promoting development and vulnerability of atherosclerotic lesions [6]. The influence of childhood exposure to several pro-inflammatory risk factors on vascular health in adult life has previously been shown, as well as the relevance of childhood exposure to cardiovascular risk factors for the later development of atherosclerosis [9–13]. Repeated bacterial or viral infections, obesity and diabetes mellitus are strong promoters of increased carotid intima-media thickness (cIMT) in children by means of chronically elevated inflammatory activity [14–16]. So far, a possible association of chronic systemic inflammation related to atopic sensitization with cIMT and other biomarkers of early vascular ageing has been analyzed mainly in adult populations [17, 18]. However, a few studies suggested that allergic diseases might contribute to early vascular ageing already in early childhood [19, 20].

Atopic sensitization, independent of its clinical penetrance, involves chronic systemic hyperinflammation. Hence, we hypothesized that atopic sensitization might contribute to early vascular ageing in young people. The primary aim of this study was to investigate a possible association of atopic sensitization, independent of its clinical significance, with the distensibility coefficient (DC) of the common carotid arteries and cIMT, which are indicators of early vascular ageing. Our second aim was to investigate whether the clinical manifestation of atopic sensitization might be associated with these parameters. Hence, we also analyzed the association of allergic disease with DC and cIMT.

## Methods

### Study population

**Fig 1:**
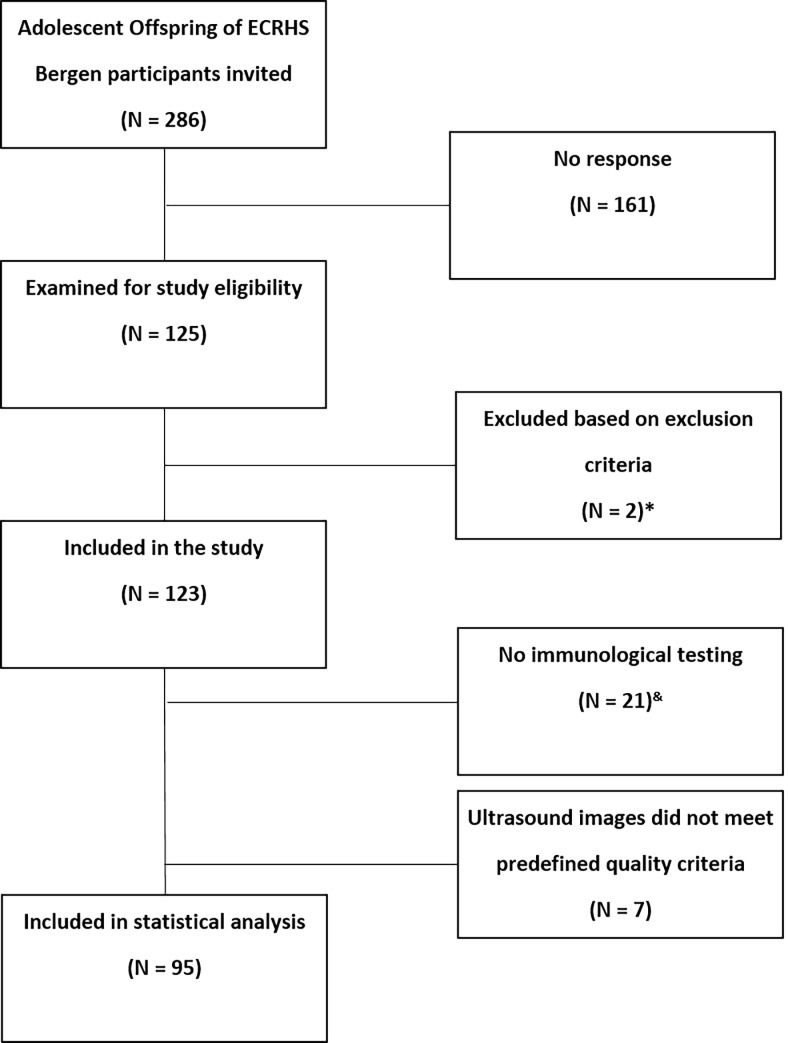
Participant recruitment process. *n = 2 with type 1 diabetes mellitus. Further predefined exclusion criteria were recent operation, an acute infection, diabetes mellitus type 2, any chronic inflammatory diseases unrelated to atopy, severe heart disease and pregnancy; ^&^No participant´s consent for blood tests.

All Norwegian offspring aged 10 to 18 years of ECRHS Bergen participants were invited to participate in the prospective RHINESSA generation study (Respiratory Health In Northern Europe, Spain and Australia, see www.rhinessa.net, Fig 1). Of the 285 offspring 125 had parental consent for clinical investigation and were screened for eligibility. Exclusion criteria were a recent operation, an acute infection, diabetes mellitus or other chronic inflammatory diseases unrelated to atopy, severe heart disease or pregnancy. Overall, we excluded two candidates because of diabetes mellitus type 1. Of the remaining 123 participants, 21 had no test of immunological total or specific IgE, because they had not agreed for blood analysis. Furthermore, seven participants’ ultrasound images did not meet the predefined quality criteria (see S1 and S2 Files for further details). Therefore, 95 participants were available for main analysis (Fig 1).

Data analysis was performed in accordance with the Declaration of Helsinki and approved by the Regional committee for Medical and Health Research Ethics, Western Region (REC West 2012/1077). Written informed consent was retrieved prior to participation (from parents if the offspring was below 16 years of age, from the offspring themselves if 16 years or older).

### Questionnaires

Extensive information on respiratory health, allergic diseases, general health and environmental exposures were assessed by a web-based questionnaire, covering all possible covariates for the analysis: parents’ atopy status, physical activity, frequent exposure to smoking (either active smoking or exposure to regular parental smoking at home), modality of birth (caesarean section versus natural birth) and preterm birth.

An interviewer-led questionnaire before clinical examination assessed respiratory symptoms during the last months and specifically during the last three days and current medication. Participants were asked to bring any regular or emergency medication to the study centers.

### Clinical examinations

Based on interview data on hours since having smoked or consumed food and drinks, medication use, and current infections, no participants were excluded from specific examinations. Afterwards, we conducted spirometry, FeNO analysis, analysis of total and specific IgE and anthropometric measures (see S1 File for detailed information).

### Main predictors: Atopic sensitization and allergic disease

Atopic sensitization was defined as a positive total or specific IgE towards inhalant allergens (house dust mite, cat, Timothy (grass, birch, and Cladosporium). Allergic disease was defined as atopic sensitization plus two of the following clinical criteria: allergic rhinitis, atopic eczema, food allergy, allergic bronchial asthma or frequent use of doctor-prescribed antihistaminic medication (see S1 File for further details).

### Ultrasonographic examination and main outcomes: DC and cIMT

ECG derived heart rate as well as systolic and diastolic blood pressure were obtained simultaneously during the ultrasonographic assessment of DC and cIMT. Blood pressure was measured on the left upper arm with an OMRON 705 IT-IS Automatic-IS device just before the examination was started and after ten minutes of rest in a sitting position. The appropriate cuff size was determined by measuring the upper arm circumference.

The procedure of DC and cIMT measurement was performed by two trained field workers, using an ultrasound instrument (UF-870, Fukuda Denshi Co. Ltd., Tokyo, Japan) with a LA38 5–16 MHz linear probe. Temporal resolution was 10.47 ms per frame. Data assessment was conducted using an automated wall-detection software, as previously described and in accordance with current recommendations to ensure acceptable data quality (see S2 File for further details; [21, 22]). In the pre-study training examinations intraobserver variability was 7.2 and 7.9%, respectively, and, thus, similar to typical values [23]. Interobserver variability was 10.7% and, thus, slightly higher than previously reported values [23]. Near- and far-wall cIMT as well as end diastolic and peak systolic outer lumen diameter were obtained from four examination planes (bilateral common carotid artery horizontal plane and ear-to-ear plane, respectively) [21]. Afterwards, a mean DC [10^−3^/kPa] and mean cIMT [mm] were calculated for each participant and used for further statistical analysis (see S2 File for further details). Validity, reliability and clinical predictive value of DC and cIMT in children and adolescents have been shown previously [24–27].

### Statistical analyses

Data analysis was performed using SPSS version 25.0 for Windows (SPSS Inc., Chicago, Illinois, USA) and R version 3.5.0 for Windows (R Foundation for Statistical Computing, Vienna, Austria). Descriptive analysis included means, standard deviations (SD), minimum and maximum values. The level of significance was set at *p* ≤ 0.05; estimated effects were reported with 95% confidence intervals (95% CI).

Unadjusted (crude) linear regression models, as well as age- and sex-adjusted multiple regression models were applied to analyze associations of DC and cIMT with atopic sensitization and allergic diseases.

Complete data were available for 95 (81.1%) of 117 participants. In the remaining 22 cases parental or participants´ consent for IgE analyses was not given. We used multiple imputation by chained equations using the “mice” package in R (version 3.0.3) to impute the missing data [28, 29]. Specifically, we imputed 300 datasets with 10 iterations each. Convergence and distribution of imputed values were assessed graphically. We applied predictive mean matching for imputation of continuous variables, logistic regression for binary variables and ordered logistic regression for ordered categorical variables [30]. The results from the regression models based on the imputed datasets were pooled using Barnard-Rubin adjusted degrees of freedom for small samples [31].

We repeated all statistical analyses with height-related standard deviation scores (SDS) of DC and cIMT, because height seems to be a strong determinant for vascular wall properties in childhood and adolescence [25]. Sensitivity analyses were performed including factors, which are more or less controversially discussed in literature as possibly being relevant for early vascular ageing and also the risk of atopic sensitization. These factors are current exposure to smoking (passive and active) [32, 33], physical activity [34], preterm birth [35] and delivery by caesarean section [36]. None of these additional analyses resulted in significantly different outcomes and, therefore, they are not presented.

## Results

### Basic characteristics of the study population

General empiric and vascular characteristic were comparable in participants with and without atopic sensitization (Table 1). Atopic sensitization was found in 33 (34.7%) of all participants with available blood samples. Of these participants, allergic disease was found in 11 (33.3%) individuals. Nine of them took antiallergic medication on a regular basis at the time of examination. Mean DC was 46.99±8.07*10^−3^/kPa in the group with atopic sensitization and 51.50±11.46*10^−3^/kPa in the group without atopic sensitization. Mean cIMT was 0.50±0.04mm in both groups.

**Table 1:** Population characteristics.

	Mean (standard deviation; min/max) / n (%)		
	*Atopic sensitization (n = 33; 34*.*7%)*	*No atopic sensitization (n = 62; 65*.*3%)*	*p-value*^*#*^
*Empiric characteristics*			
**Age [years]**	15.3 (±1.7; 12.4/18.5)	15.1 (±2.7; 9.8/18.7)	ns
**Female**	16 (48.5%)	32 (51.6%)	-
**Height [cm]**	169 (±9.2; 148/189)	166 (±13.7; 130/188)	ns
**BMI [kg/m**^**2**^**]**	21.4 (±3.3; 16.0/28.7)	21.0 (±4.1; 14.7/38.2)	ns
**BMI-SDS**^&^ [37]	0.58 (±1.23; -1.24/3.27)	0.43 (±1.64; -2.48/6.97)	ns
*Vascular characteristics*			
**DC [10**^**−3**^**/kPa]**	46.99 (±8.07; 28.79/63.97)	51.50 (±11.46; 29.30/95.83)	ns
DC-SDS*[25]	-0.77 (±0.62; -2.37/0.27)	-0.48 (±0.78; -2.51/2.26)	ns
**cIMT [mm]**	0.50 (±0.04; 0.42/0.59)	0.50 (±0.04; 0.42/0.62)	ns
cIMT-SDS*[25]	2.30 (±0.88; 0.40/4.19)	2.31 (±0.85; 0.69/4.53)	ns
**BP**_**sys**_ **[mmHg]**	108 (±8.1; 92/123)	107 (±8.4; 91/128)	ns
**BP**_**dia**_ **[mmHg]**	58 (±4.7; 49/73)	62 (±6.5; 52/90)	0.01
**Heart rate [bpm]**	68 (±19.2; 48/143)	69 (±18.8; 45/170)	ns

BMI = Body mass index; BMI-SDS = Standard deviation score of BMI; DC = Distensibility coefficient of the common carotid arteries; DC-SDS = Standard deviation score of DC; cIMT = Carotid intima-media thickness; cIMT-SDS = Standard deviation score of cIMT; BP_sys_ = Blood pressure, systolic; BP_dia_ = Blood pressure, diastolic.

^**#**^T-test derived differences between subpopulations with and without atopic sensitization were considered significant, if two-tailed p ≤ 0.05 (ns = p > 0.05).

^&^Norwegian reference population.

*Mainly European reference population.

### Association of atopic sensitization with biomarkers of early vascular ageing

Neither crude comparison of DC and cIMT, nor the age- and sex-adjusted multivariate regression model, indicated a significant association of atopic sensitization with these parameters (Table 2). However, DC tended to be lower in participants with atopic sensitization than in those without.

**Table 2:** Multiple linear regression analysis of associations of atopic sensitization and allergic disease with DC and cIMT.

Model		β	lower 95% CI	upper 95% CI	*p*
Atopic sensitization^&^	DC crude	-3.10	-8.80	2.60	0.28
	DC adjusted*	-3.04	-7.99	1.90	0.22
	cIMT crude	0.004	-0.01	0.02	0.66
	cIMT adjusted*	0.004	-0.01	0.02	0.67
Allergic disease^#^	DC crude	-1.94	-10.42	6.53	0.64
	DC adjusted*	-1.01	-8.89	6.87	0.80
	cIMT crude	0.009	-0.02	0.04	0.54
	cIMT adjusted*	0.008	-0.02	0.04	0.60

^&^Atopic sensitization = any positive total or specific IgE in the serological analyses (dermatophagoides, cat, birch, timothy grass, cladosporium)

^#^Allergic disease = atopic sensitization plus two of the following clinical criteria: allergic rhinitis, atopic eczema, food allergy, allergic bronchial asthma or frequent use of doctor-prescribed antihistaminic medication

*Models adjusted for age and sex. DC = Distensibility coefficient of the common carotid arteries; cIMT = Carotid intima-media thickness; β = Estimated effect; CI = Confidence interval; level of significance set at p ≤ 0.05.

### Association of allergic disease with biomarkers of early vascular ageing

Neither crude comparison of DC and cIMT, nor the age- and sex-adjusted multivariate regression model, indicated a significant association of allergic disease with DC and cIMT (Table 2).

## Discussion

Mean DC tended to be lower in participants with atopic sensitization than in those without. However, atopic sensitization revealed no significant association with DC and cIMT in this study population of Norwegian adolescents. Further, no significant associations of clinically apparent allergic diseases with DC and cIMT were identified.

In 2015, Evelein et al. found an increased cIMT in five-year old children with several clinical forms of allergies but no changes in arterial distensibility and elasticity [20]. They concluded that allergies are associated with arterial changes in young children. However, our data do not support their cIMT findings. Possibly, age as well as timing and severity of clinical manifestations of atopy might play a role. Yet, the tendency towards a lower DC in our participants with atopic sensitization might be a very early sign for a chronic subclinical impact of atopic sensitization on vascular ageing. Helpful markers of the severity of systemic inflammatory activity (i. e. oxLDL, high-sensitivity C-reactive protein, soluble interleukin-2 receptor, eosinophil cationic protein) were not available in both studies. However, we analyzed total and specific IgE, which are valid markers for qualitative assessment of atopic sensitization but do not give information about the severity of systemic inflammatory activity [38]. We assume that the inflammatory activity in our study population might have been somewhat heterogeneous, which would explain the lack of association of atopic status and allergic disease with DC and cIMT. Whether the strength of such an association might depend on the severity of systemic inflammatory activity needs to be investigated in larger prospective studies including more information about current and cumulative lifetime systemic inflammatory activity.

Another study found an adverse influence of repeated episodes of common childhood infectious diseases on cIMT [15]. This has also been suspected by Liuba et al. in 2005 [9]. Their multiple hit theory states that repeated episodes of acute infections might enhance oxidized modification of LDL, which plays an important role in the development of atherosclerosis by fostering foam cell accumulation and subsequent thickening of the vascular wall [39, 40]. This association of inflammation and formation of atherogenic oxidized LDL (oxLDL) has been described in autoimmune disorders as well and a comparable pathomechanism is imaginable in allergic diseases [2, 3]. Acute allergic reactions cause IgE-triggered mast cell activation and an acute-phase reaction, both leading to increased oxidative stress [2, 18, 41]. Activated mast cells degranulate cytokines, leukotrienes, prostaglandins and histamine, leading to endothelial activation and facilitated intracellular penetration of LDL [41]. Endothelial oxidative modification of LDL might be enhanced in children with allergic diseases, due to increased oxidative stress and decreased antioxidative capacity [2, 42]. Furthermore, several authors suggested that IgE-triggered mast cell activation during acute allergic reactions might lead to facilitated presentation of LDL to macrophages subsequently enhancing formation of foam cells [43, 44]. It was also suggested, that atherogenic complexes of CRP and oxLDL with or without β2-glycoprotein-I might be present during acute phase reactions [2, 3, 45, 46]. Accordingly, the severity of clinical penetrance of atopic sensitization in terms of cumulative lifetime load of number and severity of allergic bouts might be a key factor to produce a relevant atopy-associated effect on vascular ageing. The results of our analyses on participants with allergic diseases do not support this hypothesis. However, we did not have information about the severity of past clinical allergic episodes in our participants and the lifetime load of antiallergic treatment. Yet, clarification of this question might be of importance for clinicians, as they would be encouraged to control allergic diseases very carefully in order to avoid adverse effects on long-term vascular health in affected children and adolescents. Future studies should therefore obtain detailed information about the number and severity of acute clinical exacerbations of allergic diseases of their participants in the past and address this question. Finally, it would be intriguing to compare the levels of oxLDL during chronic and acute hyperinflammatory activity in atopic children and adolescents and investigate their association with accelerated arterial stiffening and increased intima-media thickness.

### Strengths and limitations

The standardized, high-quality measurements of DC and cIMT in a cohort of adolescents was a main strength of our study. Although early structural changes due to chronic systemic inflammation have been reported in children and adolescents [15, 20, 47], one should be aware, that the assessment of functional instead of structural alterations (e.g. by flow-mediated dilation), might lead to earlier detection of increased vascular risk related to atopic sensitization. Furthermore, our study does not allow establishing causality, due to its cross-sectional nature. Yet, there was a tendency towards an association of atopic sensitization with decreased DC and the non-significance of the results might be, at least in part, due to the relatively small sample size. We performed analysis of total and specific IgE, which are qualitative markers of the degree of atopic sensitization on the immunological level [38]. Further, based on interview and clinical measurements, our cohort had a relatively thorough phenotyping with regard to allergy. Further detail about the number and severity of clinical episodes of allergic disease, earlier medical treatment and assessment of further inflammatory markers (i. e. oxLDL, high-sensitivity C-reactive protein, soluble interleukin-2 receptor, eosinophil cationic protein) would have been helpful for a characterization of the lifetime load of hyperinflammatory activity in our study population. This may be especially relevant, as the relatively small number of participants with symptomatic allergic disease might be a major limiting factor for the non-significance of our results. Future studies should aim towards a detailed characterization of the extent and severity of inflammatory activity in their participants, as well as a larger sample size. A selection bias due to the exclusion of the 21 participants, due to denied parental consent for blood tests, seems also very unlikely, as their empirical, vascular and clinical allergy characteristics were not different from those included in the study.

### Conclusions and perspectives

To the best of our knowledge, this study is the first to analyze a possible association of atopic sensitization and allergic diseases with DC and cIMT in adolescents. Whereas evidence points towards an impact of systemic hyper-inflammation due to atopic sensitization on the vascular endothelium, our results do not support this assumption in adolescents [2, 3, 9, 15, 20, 39]. Better knowledge about the impact of the clinical character, main determinants and potential role of disease control of either atopic sensitization or allergic diseases might be valuable. Further studies should investigate, whether the number and severity of repeated acute clinical bouts of allergic diseases might be a predictor of early vascular ageing, rather than chronic low-grade inflammatory activity mediated by atopic sensitization.

## Supporting information

S1 File Definition of “atopic sensitization” and “allergic disease”, anthropometric and physical activity measures. (PDF)

S2 File Calculation of “DC” and “cIMT”. (PDF)

S1 Dataset (SAV)
