# Supplementary material for: Is atopic sensitization associated with indicators of early vascular ageing in adolescents?
Source: PLoS One. 2019 Aug 15;14(8):e0220198. doi: 10.1371/journal.pone.0220198 (PMC6695156; doi:10.1371/journal.pone.0220198)
Supplement: S1 File — (PDF) [file pone.0220198.s001.pdf]

Supplementary materials 1:

Definition of atopic sensitization.

Atopic sensitization was defined as any positive total or specific IgE in the serological analyses.

IgE analyses: The cut-offs were 200 kU/L and 17.5 kU/L for total IgE and specific IgE levels (dermatophagoides, cat, birch, timotei, cladosporium), respectively (1-3).

Definition of allergic disease.

Allergic disease was defined as atopic sensitization plus two of the following clinical criteria: allergic rhinitis, atopic eczema, food allergy, allergic bronchial asthma or frequent use of doctor-prescribed antihistaminic medication.

These clinical criteria were assessed by the web-based and pre-examination questionnaires and during physical examination.

Allergic rhinitis: *Watery or itchy eyes, running or stuffy nose near animals, in houses where there is a lot of dust, near trees, grass, flowers or when there is a lot of pollen around.*

Atopic eczema: *Recurrent rashes at typical locations.*

Food allergy: *Vomiting, nausea, headache, rash or shortness of breath after ingestion of food (excluding gluten- and lactose-containing food).*

Allergic bronchial asthma: Allergic bronchial asthma was confirmed, if there was evidence for a bronchial asthma and either clinical symptoms of allergy or ***FeNO*** > 35 ppb (4). Thereby, FeNO was obtained by two previously trained fieldworkers, with a NIOX MINO

device (Circassia Ltd., Oxford, UK) and according to current guidelines (5). Bronchial asthma was confirmed, if it was doctor-diagnosed before, if the participant takes anti-obstructive medication, if there were clinical signs of pulmonary obstruction or if the spirometry showed an airflow obstruction. Thereby, spirometry was performed with an Ndd-EasyOne spirometer and interpreted according to current guidelines (6). Airflow obstruction was confirmed, if $FEV_1/FVC < 0.7$ (7). If the spirometry showed a borderline result (FEV_1/FVC 0.7-0.8), airflow obstruction was confirmed only when there were correlating clinical signs of pulmonary obstruction.

Anthropometric assessment.

Height was measured with a Harpenden stadiometer and reported to the nearest complete one cm. Weight was measured to the nearest complete one kg with a digital scale. Body mass index (BMI) was calculated as kilogram body weight per square meter body surface.

Definition of physical activity level.

Physical activity was assessed via the web-based questionnaire. The adolescents had to report the hours of strenuous physical activity per week in addition to two to three hours at school. Physical activity level was defined as high, if the participant indicated 5 or more hours, thus meeting the recommended amount of physical activity by the WHO of one hour strenuous physical activity per day (8). Otherwise physical activity level was defined as low.

References:

1. Dodig S, Richter D, Benko B, Zivcic J, Raos M, Nogalo B, et al. Cut-off values for total serum immunoglobulin E between non-atopic and atopic children in north-west Croatia. Clin Chem Lab Med. 2006;44(5):639-47.

2. Lindberg RE, Arroyave C. Levels of IgE in serum from normal children and allergic children as measured by an enzyme immunoassay. J Allergy Clin Immunol. 1986;78(4 Pt 1):614-8.

3. Sicherer SH, Wood RA, American Academy of Pediatrics Section On A, Immunology. Allergy testing in childhood: using allergen-specific IgE tests. Pediatrics. 2012;129(1):193-7.

4. Brody DJ, Zhang X, Kit BK, Dillon CF. Reference values and factors associated with exhaled nitric oxide: U.S. youth and adults. Respiratory medicine. 2013;107(11):1682-91.

5. Dweik RA, Boggs PB, Erzurum SC, Irvin CG, Leigh MW, Lundberg JO, et al. An official ATS clinical practice guideline: interpretation of exhaled nitric oxide levels (FENO) for clinical applications. American journal of respiratory and critical care medicine. 2011;184(5):602-15.

6. Miller MR, Hankinson J, Brusasco V, Burgos F, Casaburi R, Coates A, et al. Standardisation of spirometry. Eur Respir J. 2005;26(2):319-38.

7. Pellegrino R, Viegi G, Brusasco V, Crapo RO, Burgos F, Casaburi R, et al. Interpretative strategies for lung function tests. Eur Respir J. 2005;26(5):948-68.

8. WHO. Global Recommendations on Physical Activity for Health. WHO Guidelines Approved by the Guidelines Review Committee. Geneva 2010. Available from apps.who.int/iris/bitstream/10665/44399/1/9789241599979_eng.pdf.
