# Supplementary material for: Is atopic sensitization associated with indicators of early vascular ageing in adolescents?
Source: PLoS One. 2019 Aug 15;14(8):e0220198. doi: 10.1371/journal.pone.0220198 (PMC6695156; doi:10.1371/journal.pone.0220198)
Supplement: S2 File — (PDF) [file pone.0220198.s002.pdf]

<u>Supplementary materials 2:</u> Calculation of DC and cIMT.

cIMT	=	Carotid intima-media thickness
DC	=	Distensibility coefficient of the common carotid arteries
oLD	=	Outer lumen diameter
RCCAH	=	Right common carotid artery horizontal plane
RCCAE	=	Right common carotid artery ear-to-ear plane
LCCAH	=	Left common carotid artery horizontal plane
LCCAE	=	Left common carotid artery ear-to-ear plane

Mean cIMT [mm]: A detailed description of the high-precision method of cIMT assessment is provided elsewhere (1, 2). In brief, a cIMT value was obtained by the automatic ultrasound detection software (Fukuda Denshi UF-870) for every heart cycle, if at least 70% of the 266 point-to-point measures at the vascular far wall in the 1cm detection window were registered at all 68-131 time points (depending on the heart rate). If cIMT was measured for at least three out of a maximum of six consecutive heart cycles, acceptable quality was approved and a plane-specific mean cIMT was calculated automatically for the respective location (RCCAH, RCCAE, LCCAH, LCCAE). A participant-specific mean cIMT was calculated by the first author out of the resulting one, two, three or four plane-specific cIMT values and used for further statistical analyses. Accordingly, each participant-specific mean cIMT value was calculated out of 37 944 – 836 304 raw values.

Mean DC [10^{-3}/kPa]: A detailed description of the high-precision method of DC assessment is provided elsewhere (1, 2). In brief, peak systolic and end diastolic oLD were obtained by the ultrasound software (Fukuda Denshi UF-870) for every heart cycle, if at least 70% of the 266 point-to-point measures at the vascular far wall in the 1cm detection window were registered at the respective time points. If peak systolic and end diastolic oLD were obtained for at least three out of a maximum of six consecutive heart cycles, acceptable quality was approved and a plane-specific mean peak systolic and end diastolic oLD were calculated automatically for the respective location (RCCAH, RCCAE, LCCAH, LCCAE). We used these plane-specific oLD values to calculate a plane-specific DC (3):

$$\mathbf{DC\ [10^{-3}/kPa] = (2 * \Delta oLD * oLD_{ed}) / (\Delta P * 0.1333) * 1000}$$

oLD_{ed} = end diastolic oLD;

ΔoLD = peak systolic oLD – end diastolic oLD;

ΔP = systolic blood pressure – diastolic blood pressure

A participant-specific mean DC was calculated by the first author out of the resulting one, two, three or four plane-specific DC values and used for further statistical analyses. Accordingly, each participant-specific mean DC value was calculated out of 1116 – 13 728 raw measurements of oLD.

References:

1. Touboul PJ, Hennerici MG, Meairs S, Adams H, Amarenco P, Bornstein N, et al. Mannheim carotid intima-media thickness and plaque consensus (2004-2006-2011). An update on behalf of the advisory board of the 3rd, 4th and 5th watching the risk symposia, at the 13th, 15th and 20th European Stroke Conferences, Mannheim, Germany, 2004, Brussels, Belgium, 2006, and Hamburg, Germany, 2011. Cerebrovascular diseases. 2012;34(4):290-6.

2. Teynor A, Caviezel S, Dratva J, Kunzli N, Schmidt-Trucksass A. An automated, interactive analysis system for ultrasound sequences of the common carotid artery. Ultrasound in medicine & biology. 2012;38(8):1440-50.

3. Doyon A, Kracht D, Bayazit AK, Deveci M, Duzova A, Krmar RT, et al. Carotid artery intima-media thickness and distensibility in children and adolescents: reference values and role of body dimensions. Hypertension. 2013;62(3):550-6.
